# Inner ear tissue preservation by rapid freezing: Improving fixation by high-pressure freezing and hybrid methods

**DOI:** 10.1016/j.heares.2014.06.006

**Published:** 2014-09

**Authors:** A. Bullen, R.R. Taylor, B. Kachar, C. Moores, R.A. Fleck, A. Forge

**Affiliations:** a Centre for Auditory Research, UCL Ear Institute, London WC1X 8EE, UK; b Laboratory of Cell Structure and Dynamics, NIDCD, National Institutes for Health, Bethesda, MD 20892-8027, USA; c Institute of Structural and Molecular Biology, Birkbeck College, London WC1E 7HX, UK; d National Institute for Biological Standards and Control, Potters Bar EN6 3QG, UK

**Keywords:** HPF, High Pressure Freezing, FS, Freeze Substitution, IHC, Inner Hair Cell, OHC, Outer Hair Cell

## Abstract

In the preservation of tissues in as ‘close to life’ state as possible, rapid freeze fixation has many benefits over conventional chemical fixation. One technique by which rapid freeze-fixation can be achieved, high pressure freezing (HPF), has been shown to enable ice crystal artefact-free freezing and tissue preservation to greater depths (more than 40 μm) than other quick-freezing methods. Despite increasingly becoming routine in electron microscopy, the use of HPF for the fixation of inner ear tissue has been limited. Assessment of the quality of preservation showed routine HPF techniques were suitable for preparation of inner ear tissues in a variety of species. Good preservation throughout the depth of sensory epithelia was achievable. Comparison to chemically fixed tissue indicated that fresh frozen preparations exhibited overall superior structural preservation of cells. However, HPF fixation caused characteristic artefacts in stereocilia that suggested poor quality freezing of the actin bundles. The hybrid technique of pre-fixation and high pressure freezing was shown to produce cellular preservation throughout the tissue, similar to that seen in HPF alone. Pre-fixation HPF produced consistent high quality preservation of stereociliary actin bundles. Optimising the preparation of samples with minimal artefact formation allows analysis of the links between ultrastructure and function in inner ear tissues.

## Highlights

• Routine high pressure freezing can preserve large depths of inner ear tissue.
• Stereocilial actin preserved by rapid freezing exhibits characteristic artefacts.
• Hybrid methods of fixation improved structural preservation of stereocilial actin.
• Improved fixation will reduce artefacts in ultrastructural studies of the inner ear.

## 1 Introduction

Preservation of biological structure in a state as ‘close to life’ as possible is best achieved by fixation of all components of the sample at the same time. From this perspective, the use of chemical fixatives is not desirable due to the time taken to diffuse fixatives through the sample and the specificity of the chemical crosslinks formed (Gilkey and Staehelin, 1986). As an alternative to such methods, rapid freeze fixation protocols have been developed and are routinely used in electron microscopy. In rapid freeze fixation, samples are cooled at a rate sufficient for water to be frozen in a vitreous state, without the formation of ice crystals (“vitrification”). Fixation occurs in milliseconds, structures are preserved hydrated, and in as close to their native state as possible. Use of rapid freezing can avoid the common perturbations to structure caused by chemical fixatives. These can include effects on tissue structure caused by shrinkage or swelling of tissue, shrinkage of cellular organelles and extraction or redistribution of cellular constituents such as lipids, proteins and DNA. In addition most chemical fixatives react with proteins and crosslink peptide chains as part of their fixative action. These reactions can be highly deleterious to epitopes and therefore compromise immunohistochemical studies. Rapid freezing can produce superior preservation of epitopes and prevent artificial aggregations of proteins and fixation and processing without the use of crosslinking fixatives can produce samples with higher antigenicity (Kellenberger, 1991, Hayat, 2000, Claeys et al., 2004). The importance of close to life preservation with minimal artefact formation has also increased with the advent of techniques such as electron tomography, which allow visualisation of three-dimensional structures at the macromolecular level (Gilkey and Staehelin, 1986, Studer et al., 2001, Studer et al., 2008, Lucic et al., 2013).

There are a variety of methods in use for rapid freeze fixation of tissues, however the depth of vitrification that can be achieved by most methods is very limited. Methods that involve plunging the sample into liquid cryogen (vitreous thin film/plunge freezing and immersion freezing) are generally limited to a few microns of vitrified sample. Penetration can be improved by methods such as cold metal block freezing (‘slam freezing’) or freezing by spraying the sample with liquid cryogen (jet freezing) but are still likely to only reach depths of a few tens of micrometres (Gilkey and Staehelin, 1986, Kachar et al., 2000). Slam freezing or immersion freezing combined with freeze fracture and deep etching have been used to produce high quality structural details of hair cells and hair bundles. For examples, details of actin arrangement in stereocilia and cuticular plate, lateral cisternae, lateral links and tip links have been examined using these methods (Hirokawa and Tilney, 1982, Forge et al., 1991, Kachar et al., 2000). However, the depth of good freezing in these methods is severely limited and using freeze-fracture it is difficult consistently to expose regions of interest and only small areas of these regions may be revealed.

Freezing larger volumes of tissue requires high pressures to be exerted on the sample, in combination with cryogenic temperatures. High pressure freezing (HPF) can extend the depth of vitrification of the sample to 200 μm (Studer et al., 1995, Studer et al., 2008). At sufficiently high pressures (210 MPa) the cooling rate required for the vitrification of water is reduced from several 100,000 K/s to a few 1000 K/s making vitrification of relatively thick samples practicable (Studer et al., 2008). Good heat conduction through the sample and between the sample and the carrier upon which it is mounted is vital for effective freezing. Air filled spaces between the tissue and the sample holder used for freezing can reduce the freezing efficiency by acting as insulators. Furthermore, air filled spaces are likely to collapse when high pressures are applied, deforming the tissue. To solve these problems, sample holders are often loaded with cryoprotectant fillers. A filler can act both as a cryoprotectant to improve freezing, and protect the sample against pressure induced shearing (Galway et al., 1995). Fillers are often substances that cannot penetrate the tissue and have low osmotic activity, for example solutions of dextran or the inert solvent 1-hexadecene. Alternatively cryoprotectants that penetrate the tissue, for example glycerol, can be used. The use of penetrating cryoprotectants can be undesirable, as their penetration into the tissue can lead to structural changes. However, in some cases this penetration seems to have no effect, probably due to diffusion barriers preventing entry to the tissue during the short exposure to the cryoprotectant (Dahl and Staehelin, 1989, McDonald et al., 2007, Studer et al., 2008).

After freezing, samples can be handled in a variety of ways and examined either at cryogenic temperatures or at room temperature after additional processing. Freeze substitution (FS) is a common technique for the low temperature dehydration of samples, before embedding in resin and examination at room temperature. Water in the samples is replaced by a solvent (usually acetone) at a temperature around −90 °C. This temperature is lower than the lowest temperature at which secondary ice crystals have been shown to form in biological samples (−70 °C) (Steinbrecht, 1985). Fixatives and heavy metal, electron dense stains such as osmium tetroxide and uranyl acetate are usually added to the sample to stabilise the frozen structure and improve image contrast. Because chemical fixation occurs when the sample is already frozen, there is no liquid water present and osmotic effects should not occur (Studer et al., 1992). Fixation by osmium tetroxide likely begins at about −70 °C (White et al., 1976). Once dehydration and infiltration of fixatives have occurred, the sample is slowly warmed to a suitable temperature for resin embedding.

Although HPF/FS protocols eliminate many of the artefacts associated with conventional fixation and embedding, the process of rapid freezing and substitution can itself cause artefactual changes to the tissue. Freezing artefacts, such as the growth of small ice crystals within the tissue due to insufficiently rapid freezing or subsequent warming are a common problem. Ice crystals formed during freezing cause dehydration in the surrounding cytoplasm, and solutes are concentrated as water is recruited to the growing ice crystal. After FS these effects are observed as characteristic holes in the sample (from the ice crystal branches) and aggregates of solute in the surrounding cytoplasm. In severe cases these changes in solute concentration can lead to the rupture of membranes (Galway et al., 1995, Dubochet, 2007). Where growth of small ice crystals has occurred a reticulated pattern is also often visible in the sample (Gilkey and Staehelin, 1986). Complex structures containing multiple different cell types such as the organ of Corti can prove difficult to vitrify uniformly due to differing freezing characteristics throughout the tissue. Often, the protocol used for HPF must be tailored specifically to the tissue in question (Koster and Klumperman, 2003).

Despite the potential advantages of freeze fixation for ultrastructural studies, there has been limited use of HPF methods for the examination of inner ear tissues. Work by Triffo et al. (2008) showed that a combination of microaspiration and HPF could be used to preserve isolated strips of guinea pig outer hair cells (OHCs) and work by Meyer et al. showed that good preservation of mammalian inner hair cells (IHCs) and particularly synaptic peripheral processes and morphology could be achieved by HPF but that consistent preservation across the organ of Corti was difficult (Meyer et al., 2009). The aim of this work was to evaluate the preservation of cells and structures in the organ of Corti and vestibular tissue of mammals and amphibians dissected and preserved by HPF and FS and to compare preservation to that achieved by conventional fixation methods. The use of hybrid methods of preservation was also examined.

## 2 Material and methods

**Animals:** Inner ear tissues were obtained from adult gerbils, adult tri-colour guinea pigs, C57/Bl6 mice at around P30, and from the red-spotted newt *Notophthalmus viridescens*. Rat tissues were obtained from postnatal or older rats as described in Dumont et al. (2001). All work with animals was conducted in accordance with procedures licenced by the British Home Office and approved by UCL Animal Ethics committee or in accordance with the National Institutes of Health (NIH) guidelines for animal care and use under protocol NIH 1215-11.

### 2.1 Experimental methods

**High Pressure Freezing:** For fresh frozen samples from gerbil and guinea pig, the auditory bullae were removed and held on ice. Cochlear and vestibular tissues were dissected from the bullae just prior to freezing. The time between sacrifice and freezing was approximately thirty minutes. Isolated mouse utricular maculae were removed to glutamax minimum essential medium (Life Technologies, UK) with HyClone serum (Thermo Fisher Scientific, USA) and 10 mM 4-(2-Hydroxyethyl)piperazine-1-ethanesulfonic acid **(**HEPES) (pH 7.3) (Sigma–Aldrich, USA) and maintained for 4–6 h prior to freezing. Otic capsules from the newts were removed from the animals into Amphibian Dulbecco's phosphate buffered saline (Taylor and Forge, 2005) and the inner ear samples consisting of the saccular maculae, amphibian papilla, and lagena macula were isolated then transferred to culture medium (Taylor and Forge, 2005) for maintenance at room temperature for 30–90 min prior to freezing.

For fixed tissue, auditory bullae were isolated and opened to expose the cochleae. Fixative was gently injected into the cochlea and vestibular system via an opening made at the apex of the cochlea and a widening into the vestibule created by breaking the bone between the round and oval windows and removing the stapes. The bulla was then immersed in fixative and fixation continued for 1.5 h at room temperature. The fixative was 2.5% glutaraldehyde (Agar Scientific, UK) in 0.1 M cacodylate buffer pH 7.3 with 3 mM CaCl_2_. Individual cochlear turns were then dissected under 0.1 M cacodylate buffer straight after the end of the fixation period and immediately prepared for high pressure freezing.

For freezing, samples were loaded into 200 μm deep aluminium planchettes, (Leica Microsystems, Germany). The planchette was filled with a cryoprotectant/filler and covered with the flat (non-depression) side of a second planchette pressed firmly down to remove air and to minimise the total depth of the freezing sample to 200 μm. Tissue from mice, gerbils and guinea pigs was frozen in a Leica HPM 100 high pressure freezer (Leica Microsystems). Newt tissue was frozen in either the Leica machine or a Bal-Tec HPM 010 high pressure freezer (BAL-TEC AG, Liechtenstein). Freezing rates in both devices were greater than 25,000 K/s.

Several different cryoprotectants and fillers were tested: 20% Dextran (Sigma–Aldrich) in 10 mM HEPES-buffered (pH 7.3) Hank's balanced salt solution (HBSS) (Sigma–Aldrich) (HB-HBSS); 1-hexadecene (Sigma–Aldrich); 25% glycerol (Sigma–Aldrich) in 0.1 M cacodylate buffer; or a yeast paste of commercial bakers yeast (Allinson, UK) reconstituted in HB-HBSS (fresh tissue) or 0.1 M cacodylate buffer (fixed tissue). All cryoprotectants were introduced to the tissue in the planchette at room temperature, directly before freezing. Time between cryoprotectant introduction and freezing was <3 min. After freezing samples were stored under liquid nitrogen until freeze substitution (FS).

**Slam Freezing**: For contact or slam freezing we used two different apparatuses. First, utricular maculae were isolated from the auditory bullae of guinea pigs under HB-HBSS and immediately transferred on to thin copper specimen supports. The sample was brought rapidly into contact with (“slammed” against) the polished surface of a copper block cooled by liquid nitrogen by a controlled bounce-suppressed drop under gravity using a “Gentleman Jim” device (Phillips and Boyne, 1984). The frozen sample attached to the specimen support was stored under liquid nitrogen until FS. During FS the sample separated from the support. Alternatively, samples were slam frozen using Life Cell CF0100 quick-freezing machine (Research and Manufacturing, USA) as previously described (Dumont et al., 2001). Briefly, rat organ of Corti was dissected in L-15 medium (Sigma) and positioned gently onto a 400-nm-thick slice of Bacto-agar (Difco Laboratories, USA) and mounted on an aluminium specimen holder. The sample was slammed against the liquid nitrogen-cooled surface of a sapphire block in the Life Cell quick-freezing machine and transferred to liquid nitrogen before freeze-substitution.

**Freeze Substitution:** FS was carried out in a Leica AFS Device (Leica Microsystems). Planchettes were opened under liquid nitrogen and then placed into processing baskets before being transferred, under liquid nitrogen, into pre-cooled solutions. Solutions were made up in acetone and temperature maintained at −90 °C during substitution. Samples were first maintained in a solution of 0.1% tannic acid and 0.5% glutaraldehyde in acetone for 24 h. This was followed by three one hour washes in acetone before transfer to 0.1% Osmium tetroxide and 1% uranyl acetate in acetone and maintenance at −90 °C for 48 h. For pre-fixed samples, glutaraldehyde was omitted. Samples were then immersed in pure acetone and the temperature was raised from −90 °C to 20 °C over 36 h, with acetone exchanges every 3–6 h. After substitution samples were removed from planchettes under acetone and embedded in the Epon resin (Agar100; Agar Scientific), following the manufacturer's instructions.

Samples slam frozen with the Life Cell machine were freeze substituted in 1.5% uranyl acetate in methanol at −90 °C, infiltrated with Lowicryl HM-20 resin at −45 °C, and polymerized with ultraviolet light as previously described (Dumont et al., 2001). The exact methodology of fixation and freeze substitution for each figure panel can be found in the supplementary methods.

**Conventional Fixation:** Following removal from the otic capsule, the inner ear tissues of the newt were fixed in 2.5% glutaraldehyde or 2% glutaraldehyde with 2% paraformaldehyde in either 1 mM or 0.08 mM cacodylate buffer or in amphibian Dulbecco's phosphate buffered saline for 2 h at room temperature before post fixation in 1% OsO_4_ in either 1 mM or 0.08 mM cacodylate buffer. Mouse inner ear tissues were dissected, fixed in 2.5% glutaraldehyde in 0.1 M cacodylate buffer pH 7.3 with 3 mM CaCl_2_ for 2 h at room temperature. After fixation tissue was decalcified by incubating at 4 °C for two days in a solution of 4% ethylenediaminetetraacetic acid (EDTA) (Sigma–Aldrich, UK) in 0.1 M cacodylate buffer. Post-fixation staining was carried out in 1% aqueous OsO_4_. Samples were dehydrated in an ethanol series, en bloc stained with a saturated solution of uranyl acetate in 70% ethanol overnight at 4 °C before completion of dehydration and embedding in plastic.

**Electron microscopy:** 80 nm sections of embedded tissue were cut using a diamond knife and collected on uncoated copper grids. To help reduce image drift during examination of the sections at high magnifications (greater than 30,000×), a thin carbon coat was applied by evaporation to the grid on top of the sections. Carbon coating was performed in a Balzers BAF 400D freeze fracture device (Balzers AG, Liechtenstein).

Sections were examined in a JEOL JEM1200EX-II transmission electron microscope (JEOL UK, Welwyn Garden City) operating at 80 kV and images collected with a Gatan Ultrascan 1000 digital camera (Gatan, USA) or on a Zeiss 922 microscope operated at 160 kV and images collected with a Gatan Ultrascan 2000 digital camera (Gatan). Digital images were adjusted for optimal contrast and brightness, and image panels were assembled into figures using Adobe Photoshop CS6 (Adobe Systems Software Ltd, Ireland). Measurements of freezing depth were obtained from images of the sections using the measuring feature of the ImageJ programme (Schneider et al., 2012).

## 3 Results

### 3.1 Cellular preservation

The preservation of inner ear tissue by HPF was assessed in a number of ways, using the tissues of different species under different freezing conditions. Specific animal models were chosen based on methodological criteria and their utility in hearing research. Mice are one of the most common animal models for inner ear disorders. However, it is difficult to dissect the unfixed cochleae of adult mice so assessment of the non-prefixed organ of Corti was made primarily with tissue from gerbils and guinea pigs whose cochleae are easier to dissect, with some additional assessment of rats. Utricular maculae can easily be isolated from mice as well as guinea pigs and gerbils, allowing testing across several mammalian species to ensure there were no species-specific differences in preservation. In addition the newt species *N. viridescens* was used to determine if HPF could overcome difficulties encountered in obtaining good preservation of inner ear tissues by conventional means. This newt model has been previously used by our laboratory (Taylor and Forge, 2005). In addition, a variety of well-established non-penetrating cryoprotectants were chosen to avoid potential artefactual changes caused by penetration of the cryo-protectant into the tissue. The quality of freezing was assessed by the presence of freezing artefacts, holes or distortions in the tissue, reticulation patterns in cytoplasm or nucleus, or changes to the cytoplasm indicating local dehydration.

**Fig. 1:**
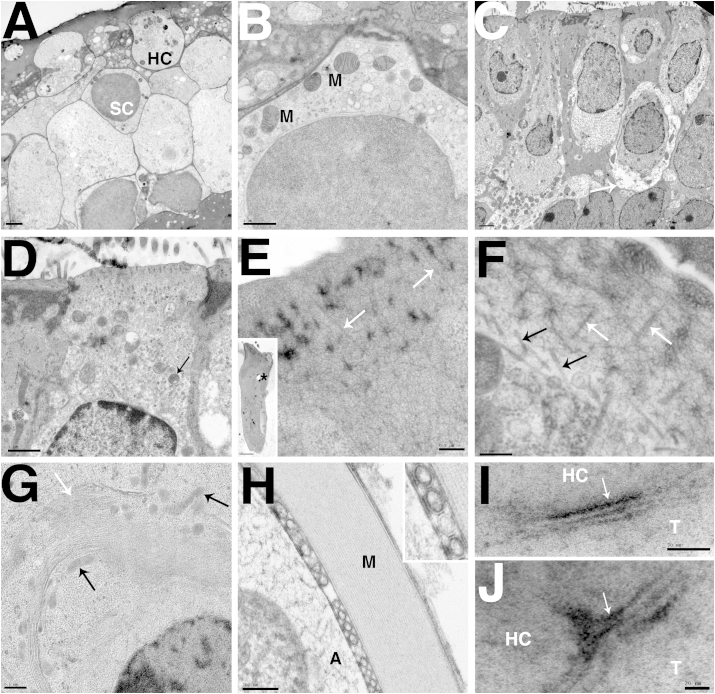
Preservation of mammalian inner ear tissue by HPF. A. Utricular macula from P30 mouse frozen in dextran cryoprotectant. There is good freezing and tissue preservation through the entire depth of the sensory epithelium with no indications of ice-crystal growth in cytoplasm or nuclei in either hair cells (HC) or supporting cells (SC). White objects in hair cells and supporting cells are unidentified, but appear to be membrane bound. B. Supporting cell in P30 mouse utricular macula frozen in dextran cryoprotectant. The cytoplasm shows an even electron density with no indication of granular “clumping” of constituents. Likewise the nucleus shows even electron density. Mitochondria (M) are well preserved and show prominent irregular cristae. Other cell organelles are visible distributed within the cytoplasm. C. Vestibular macula from adult mouse conventionally fixed with glutaraldehyde. Cells show typical artefacts from chemical fixation and dehydration including cell shrinkage and granular cytoplasm when compared to rapidly frozen cells in (A). Spaces around the cells (white arrow) were often observed. D. Hair cell from vestibular macula from adult mouse conventionally fixed with glutaraldehyde. Granular cytoplasm and granular uneven electron density of the nucleus can be observed. Some mitochondria (black arrow) showed uneven electron density. E. Cuticular plate of OHC from adult guinea pig organ of Corti frozen in yeast paste. The fibrillar nature of structure of the cuticular plate is evident. Stereociliary rootlets are indicated (white arrows) and show fibrillar structures extending into the cuticular plate. Inset shows a complete OHC, showing even cytoplasm and no evidence of ice crystal damage, throughout the cell. The hole (*) was caused by sectioning and is not a freezing artefact. F. Cuticular plate from P30 mouse utricle frozen in dextran cryoprotectant. White arrows indicate stereociliary rootlets that show crosslinks with well-preserved fibrils within the cuticular plate. Microtubules in the apical cytoplasm underlying the cuticular plate are clearly resolved (black arrows). G. IHC from adult guinea pig organ of Corti frozen in yeast cryoprotectant. Preservation of the fine structure of internal cell membranes (white arrow) and mitochondria showing cristae and even electron density (black arrows) are indicated. H. Myelinated axon from adult guinea pig cochlea frozen in hexadecene cryoprotectant. Myelin sheath (M) and cytoplasm of axon (A). The myelin layers are closely parallel with no indication of distortion due to ice crystal growth. Inset shows regularly arranged membranous structures observed in the extraplasmalamellar space of the axon. I and J: Vestibular synapse from adult gerbil utricle frozen in dextran cryoprotectant. Hair cell (HC) and synaptic terminal (T) are indicated. Presynaptic density is indicated (white arrow) and synapses showed close membrane apposition between the terminal and hair cell and internal structure. Scale Bar: A: 2 μm, B: 1 μm, C: 2 μm, D: 1 μm, E: 0.5 μm (Inset 2 μm) F: 200 nm, G: 0.5  μm H: 200 nm, I: 50 nm, J: 20 nm.

**Fig. 2:**
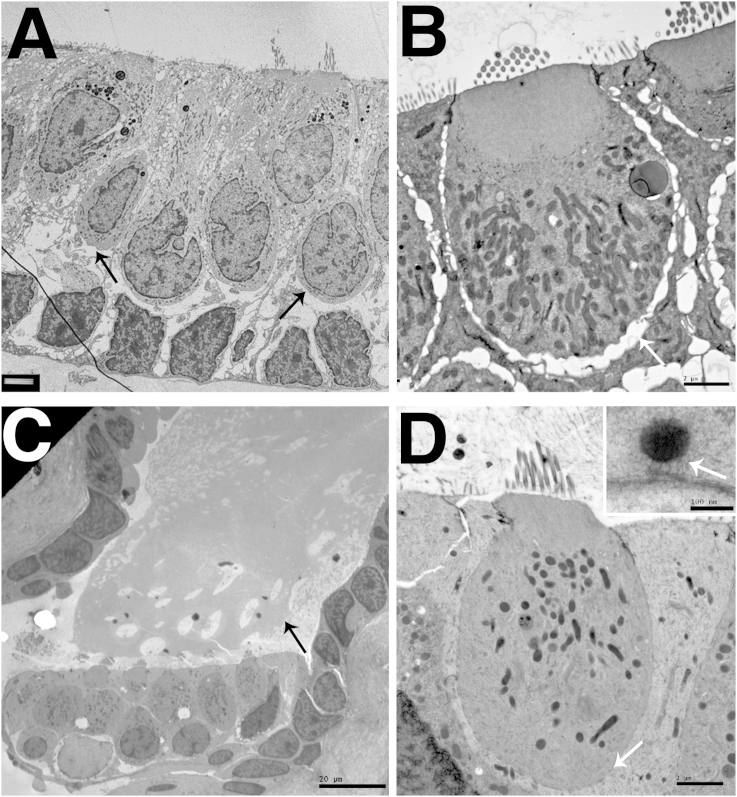
Comparison between HPF and conventional fixation of adult newt sensory epithelia. A–B: conventional fixation. C–D: High-pressure frozen in dextran cryoprotectant and freeze-substituted. A. Saccular macula. Large extracellular spaces appear in conventionally fixed tissue with shrunken supporting cells (black arrows). The nerve endings are retracted. B. Hair cell in amphibian papilla surrounded by extracellular spaces that appear to result from shrinkage of supporting cells and retraction of nerve (white arrow). C. Amphibian papilla after HPF/FS. The entire depth of the amphibian papilla is well frozen and the tectorial membrane is in place and shows no indication of shrinkage (black arrow). D. Individual hair cell in amphibian papilla is well preserved and supporting cells closely appose the cell body of the hair cell (white arrow). Inset shows ribbon synapse from newt hair cell, white arrow indicates preservation of structure tethering the ribbon to the synapse. Scale bars: A: 0.5 μm, B: 2 μm, C: 20 μm, D: 2 μm (Inset: 100 nm).

Cellular preservation in fresh frozen tissue from all of the tested species was generally good, with limited freezing artefacts. Damage was observed in some samples, mostly in the form of holes in the tissue that may have been caused by ice crystals, and reticulation in the nuclei of some cells. However, such artefacts were not widespread in the tissue. Good preservation was shown through large depths of tissue (Fig. 1, Fig. 2d) and was achieved in samples from mouse (Fig. 1a, b, f), newt (Fig. 2), gerbil (Fig. 1i and j), and guinea pig (Fig. 1e, g, h) with some variation of the freezing conditions. Dextran was used as a cryoprotectant in the gerbil, newt and mouse samples, but in guinea pig hexadecene (Fig. 1h) or yeast (Fig. 1e, g) were used. In fresh frozen samples, significant differences in preservation between different cryoprotectants were not observed. Excellent preservation of nuclei without reticulation was seen and cytoplasm was generally smooth (Fig. 1a, b, e–g). In comparison with similar conventionally fixed samples in tissue preserved by HPF cells showed even, non-granulated cytoplasm and nuclear contents without the ‘clumping’ and granularity that is often an artefact of chemical fixation (Caulfield, 1957). There was no evidence of cell deformation by shrinkage, cell membranes were closely apposed and there were no spaces around cells (Fig. 1a, b and Fig. 2c, d) unlike conventionally fixed samples (Fig. 1, Fig. 2a,b). At higher magnification, uneven electron density in conventionally fixed mitochondria was also observed, HPF fixation produced mitochondria with even density (Fig. 1b and d). Preservation was good throughout cochlear hair cells (Fig. 1e inset). Cuticular plate structure in both cochlear and vestibular hair cells was well preserved, and the fine fibrillar links between stereociliary rootlets and the actin mesh were always observed. The vestibular hair cell also showed excellent preservation of the microtubules underlying the cuticular plate and the network did not appear disturbed by ice crystal formation (Fig. 1e, f). Internal membranes showed no evidence of swelling or distortion by ice crystals, mitochondria and other organelles in both hair and supporting cells were also extremely well preserved (Fig. 1b, g). There was also very good preservation of myelinated axons, these exhibited parallel protein layers in the myelin, without the shrinkage often seen after chemical fixation or distortion from ice crystal formation. Axons preserved by this method often showed regularly arranged membranous structures in the extraplasmalamellar space (Fig. 1h). Good preservation of IHC synapses has already been shown (Meyer et al., 2009). Vestibular synapses were also well preserved, showing close apposition of cell membranes with no shrinkage of the terminal and evidence of internal structure of the synapse both in gerbils (Fig. 1i and j) and in newt tissue (Fig. 2d (inset)).

### 3.2 Preservation of innervation in sensory epithelia from the newt

HPF fixation of newt tissue showed that similar well-preserved samples could also be achieved in non-mammalian species. The value of rapid freezing by high pressure in comparison with conventional fixation for structural preservation was particularly apparent in preparation of inner ear tissue from the newt. In conventionally fixed samples, large extracellular spaces were present within the sensory epithelia (Fig. 2a). Hair cells appeared quite well preserved but spaces developed around the bodies of the supporting cells, which appeared shrunken, and neuronal elements were largely absent; often only remnants of ruptured nerve endings remained (Fig. 2b). Efforts to better preserve the tissue by altering osmolality of the fixative solution, the pH and nature of the buffer in which the fixative was diluted, the concentration of fixative and the nature of the fixative (glutaraldehyde or glutaraldehyde-paraformaldehyde) all failed to prevent the development of the spaces and the loss of the neuronal elements (data not shown). Hence although the relatively well-preserved hair cells showed the presence of ribbon synapses in hair cells of the amphibian papilla, post-synaptic specialisations were often difficult to assess.

In contrast, in high pressure frozen samples that had not been exposed to chemical fixatives (fresh frozen tissue), inner ear epithelia were compact and showed no evidence of enlarged extracellular spaces or shrunken supporting cells (Fig. 2c). The overlying extracellular matrices, such as the tectorial membrane of the amphibian papilla showed no indications of the shrinkage and displacement that is normally seen with conventional fixation (Fig. 2c arrow). The innervation and the synapses at the base of the hair cells were also well preserved (Fig. 2d), as was the fine detail of structures at the synapse (Fig. 2d (inset)). The marked differences between the two preparation protocols underlines the potential importance of rapid fixation in preserving the morphology of inner ear tissues.

### 3.3 Stereocilia preservation: HPF-derived artefacts

**Fig. 3:**
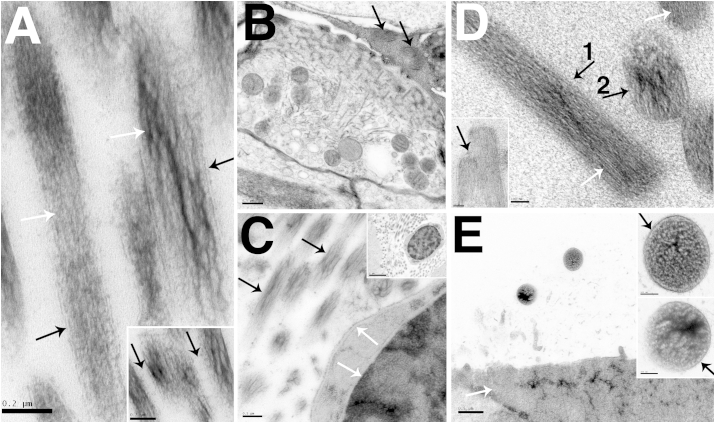
Preservation of stereociliary actin by HPF. A. Stereocilia from P30 mouse utricle frozen in hexadecene cryoprotectant. Actin fibrils are tangled (white arrows). But the surrounding stereociliary membranes are mostly smooth (black arrows) showing no ice crystal induced distortions. Inset shows intact interstereocilial links (black arrows). B. Stereocilia and hair cell from P30 mouse utricle frozen in dextran cryoprotectant. Poorly preserved stereocilia actin (black arrows) is shown in context with the well-preserved hair cell cytoplasm. C. Stereocilia from adult gerbil utricle frozen in dextran cryoprotectant. Good cellular preservation, shown by the lack of reticulation in the nucleus, smooth cytoplasm and well-preserved plasma membrane (white arrows) is seen in context with tangled stereocilia actin and smooth stereocilia membrane (black arrows). Inset shows low power view of the same area. D. Stereocilia from P30 mouse utricle frozen in dextran cryoprotectant. Tangled actin (black arrow 1) and tangled actin with smooth stereocilia membrane (black arrow 2) are both present. Small areas of parallel actin were also observed (white arrows). Inset shows mouse utricle stereocilia with parallel actin. The tip link is also preserved (black arrow). E. IHC in adult guinea pig cochlea frozen in yeast cryoprotectant. Cuticular plate (white arrow) is well preserved showing fibrillar details. In the stereocilia, shown at higher power in insets, while the membrane is smooth (black arrows), the internal actin structure is disrupted. Scale Bar: A: 0.2 μm (inset: 0.2 μm), B: 0.5 μm, C: 0.5 μm (Inset: 2 μm), D: 100 nm (Inset 100 nm), E: 0.5 μm (Insets: 100 nm).

While the preservation of cytoplasmic structures and organelles in samples prepared using HPF was generally very good, that of stereocilia was often poor. The actin core of the stereocilia had a ‘tangled’ appearance after freezing. The normally parallel actin strands were distorted, giving the appearance of areas of high electron density and the strands in the core of a stereocilium were not parallel (Fig. 3a). Tangled stereociliary actin was observed in all the samples tested, regardless of species (including the newt) or the cryoprotectant used when freezing the tissue. Tangled actin was often observed close to areas of good cellular preservation (Fig. 3b and c). Despite the tangled actin, the stereociliary membranes appeared to be smooth in both longitudinal and transverse sections, showing no evidence of distortion due to ice crystal formation (Fig. 3a,c,d and e). However, even in samples where most of the stereocilia showed this tangled actin, there were occasional stereocilia containing parallel actin strands or regions of good actin preservation in stereocilia with otherwise tangled actin (Fig. 3d). Crosslinks between stereocilia were evident, even where the actin filament bundles were distorted (Fig. 3a inset) and tip-links were also present (Fig. 3d) both in regions where there were parallel actin filaments and in those where the filaments were tangled (data not shown).

The abnormalities of the stereocilia could be due to the effects of high pressure in HPF or the result of local variations of freezing rates in the tissue. These variations would have to be very small to account for the observation of poorly preserved stereocilia in otherwise well preserved tissue. Such local variations in freezing rates could also account for the observation of occasionally well-preserved stereocilia in the samples and previous accounts of well-preserved actin in rapidly frozen stereocilia (Hirokawa and Tilney, 1982, Dumont et al., 2001, Rzadzinska et al., 2004).

**Fig. 4:**
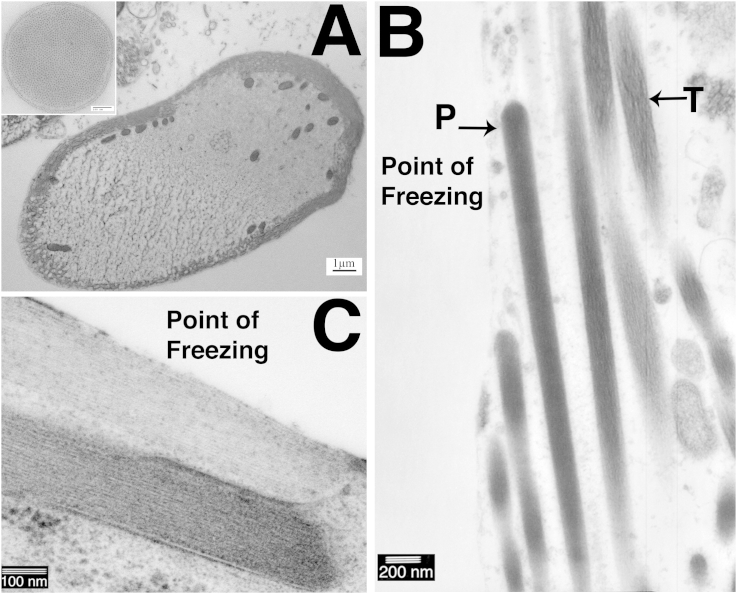
Slam frozen preparations. Adult Rat Organ of Corti (A). Adult Guinea pig utricle (B,C). A. Rapid deterioration in the quality of freezing with distance from the freezing front in an OHC in a sample slam frozen at liquid nitrogen temperature: approximately half the cell width is well frozen with even cytoplasm but abrupt change to “granular” appearing cytoplasm and disruption of the lateral cisternae due to distortions resulting from ice-crystal growth. A (inset): Cross section of stereocilium in sample slam-frozen at liquid nitrogen temperature shows the excellent preservation of actin bundle structure possible with this method. B. Parallel actin filaments are observed close to the freezing front (P) but preservation of actin structure deteriorated moving away from the freezing front and actin filaments became tangled (T). C. Parallel actin filaments close to the freezing front in longitudinal sections. Compression of stereocilia can also be observed. Scale Bars: A: 2 μm (inset 100 nm), B: 200 nm, C: 100 nm.

To explore whether the tangled actin was the consequence of the high pressures in HPF or an effect of freezing, unprefixed samples were prepared by an alternative freezing technique often used to examine inner ear tissues, slam freezing. This method can produce excellent preservation of the filamentous structure of stereocilia (Fig. 4a (inset)). However the depth of good freezing is limited, as seen in a cross-section of an OHC where the quality of freezing can be seen to deteriorate dramatically across the width of the cell, ice crystal damage being evident over at least half of the cytoplasm (Fig. 4a). In a hair bundle from a guinea pig utricular macula, used as an example since tangled actin was seen in samples from all species but in which hair bundles are large and numerous, it was found that the stereocilia closest to the freezing front exhibited parallel filaments, and appeared well preserved, while those further away showed tangled actin morphologically identical to that observed in samples frozen by HPF (Fig. 4b). This indicates that the disruption of the actin filaments is most likely a consequence of local variations in freezing rates. The sample in Fig. 4a was freeze substituted without tannic acid and osmium tetroxide, but no significant differences were observed between these samples and those substituted with tannic acid and osmium tetroxide present (Fig. 4b and c).

In the slam-frozen samples, tangled actin was usually observed within 1 μm of the freezing front. Gross freezing damage to the whole sample, significant enough to be observed at low power magnifications, occurred within 9 μm of the freezing front (data not shown). Nevertheless, in those areas close to the freezing front preservation of actin bundles in stereocilia was often extremely good, showing parallel filaments as previously described. However, in some samples compression artefacts resulting from the slam freezing could be observed, such as pushing together of stereocilia (Fig. 4c). Slam-frozen samples showed good preservation of the stereociliary membrane, and an even spacing between the membrane and actin bundle. High quality preservation of actin filament bundles in stereocilia was therefore possible by rapid freeze fixation, but consistent preservation throughout a sample could not be achieved.

### 3.4 Stereocilia preservation: hybrid methods

**Fig. 5:**
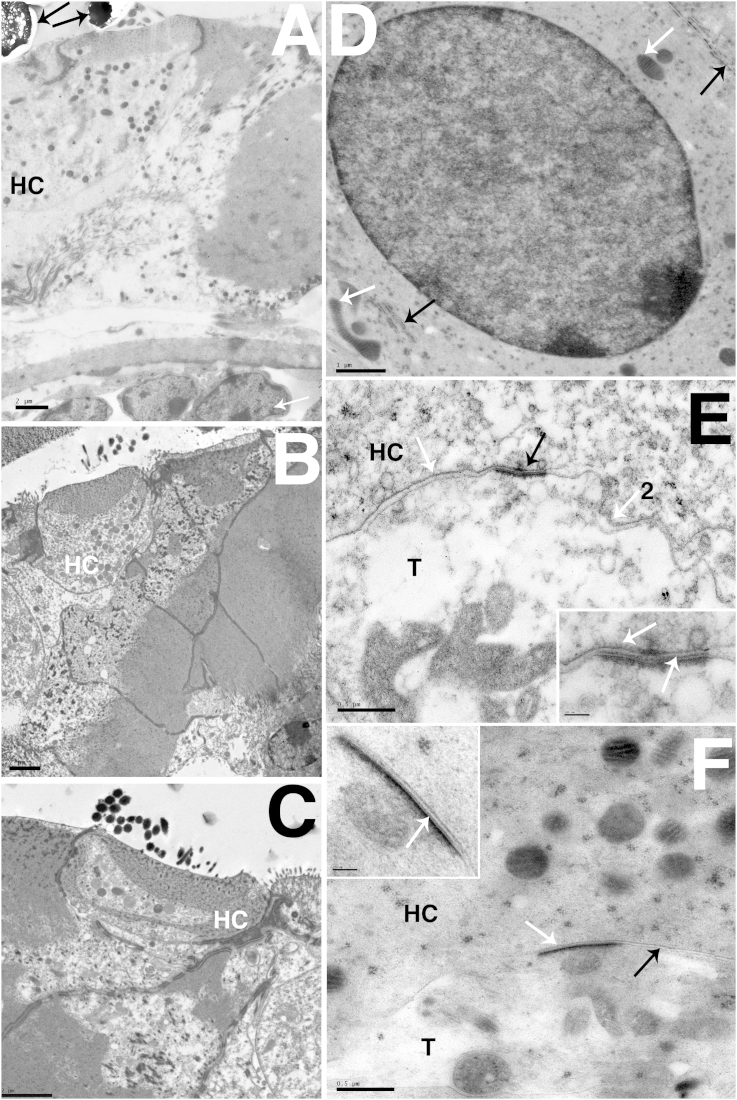
Preservation of Adult Guinea Pig cochlea by pre-fixation and HPF. A. Pre-fixed HPF frozen organ of Corti frozen with yeast paste cryoprotectant. IHC cytoplasm (HC) and that of surrounding cells was smooth and consistently preserved without granular appearance. Good freezing extended throughout the tissue and no freezing artefacts can be seen in nuclei of cells underlying the basilar membrane (white arrow). Black arrows indicate defects in the resin caused by non-infiltration of the yeast cells, and are not defects in the tissue. B. Pre-fixed organ of Corti frozen with glycerol cryoprotectant. Unlike the cytoplasm of HPF fixed and prefixed cells frozen in yeast paste, IHC cytoplasm (HC) and cytoplasm of surrounding cells showed a distinct granular appearance. C. Pre-fixed organ of Corti frozen with hexadecene cryoprotectant. Similar to the glycerol cryoprotectant the cytoplasm of IHCs (HC) and surrounding cells had a granular appearance. D. Nucleus of an IHC preserved by pre-fixation and HPF with yeast paste. Nucleus shows no obvious artefacts from either freezing or chemical fixation. Mitochondria with even density and well-defined cristae (white arrows) and rough endoplasmic reticulum with no evidence of luminal shrinkage or swelling (black arrows) are also shown. E. IHC synapse preserved by pre-fixation and HPF with glycerol. Synapse (black arrow) and apposition of IHC (HC) and synaptic terminal (T) membrane (white arrow) is shown. Both IHC and terminal cytoplasm show uneven density and the terminal membrane shows some shrinkage from the hair cell membrane (white arrow 2). High magnification of synapse shows structure on the presynaptic membrane (white arrow) and within the synaptic gap (black arrow). F. IHC synapse preserved by pre-fixation and HPF with yeast paste. Apposition of IHC (HC) and synaptic terminal (T) membrane (black arrow) is closer than that observed in the sample preserved with glycerol and showed no evidence of shrinkage and cytoplasm has even density. Synapse (white arrow in main panel and inset) showed close apposition of membranes and some evidence of internal structure. Scale Bars: A–C: 2 μm, D: 1 μm, E–F: 0.5 μm (inset 100 nm).

**Fig. 6:**
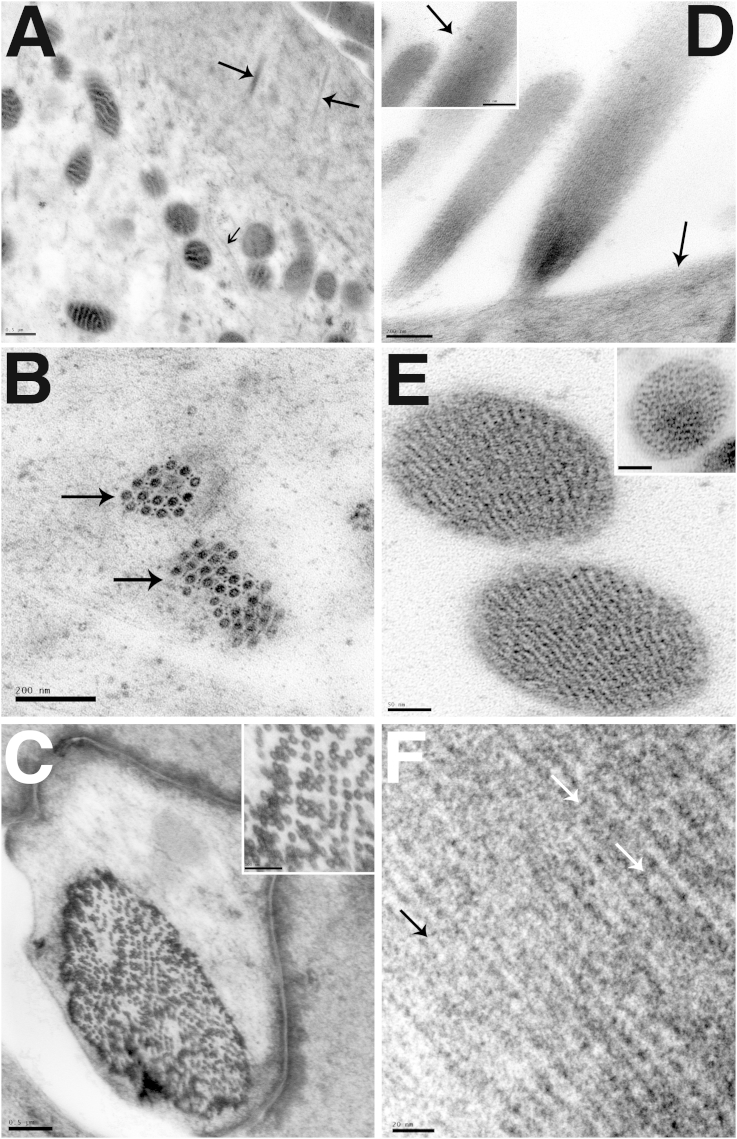
Preservation of Adult Guinea Pig cochlea by pre-fixation and HPF – Apical Structures. A. Cuticular plate and cytoplasm of an IHC frozen in the presence of yeast paste. Stereocilia rootlets (closed head arrows) crosslinked to fibrils in the body of the cuticular plate. Microtubules in the apical cytoplasm beneath the cuticular plate are well preserved (open headed arrow). B. Microtubule bundles (arrows) from a yeast paste frozen IHC. Structure between the microtubules can be visualised. C. Deiters' cell phalangeal process (P) frozen with hexadecane. The organisation of microtubule bundles (M) is well preserved as shown at higher magnification in the inset. D. IHC stereocilia frozen with yeast paste. Parallel actin and a well-preserved cuticular plate (arrow) can be seen. Inset shows tip-link between stereocilia (arrow). E. Transverse stereocilia sections showing the pattern of actin. Tilted sections show a pattern of parallel lines and a straight section (inset) shows a circular pattern. Frozen with yeast paste. F. Stereociliary actin showing actin crosslinking (arrows). Frozen with yeast paste. Scale Bars: A: 0.5 μm, B: 200  nm C: 0.5 μm, D: 200 nm (inset 200 nm), E: 50 nm (inset 50 nm), F: 20 nm.

While rapid freeze fixation aims to eliminate chemical fixatives and potential consequent artefactual changes to the tissue, it has been shown previously that pre-fixation of tissue with aldehydes before HPF can be beneficial in preserving highly labile and delicate structures (Meissner and Schwarz, 1990, Sosinsky et al., 2005, Sosinsky et al., 2008). Glutaraldehyde was chosen as a prior fixative due to its superior preservation of fine ultrastructural detail and its use in prior studies of pre-fixation before freezing (Meissner and Schwarz, 1990, Sosinsky et al., 2008). To test whether pre-fixation could improve the preservation of actin in stereocilia during HPF; samples of guinea pig organ of Corti were fixed in glutaraldehyde and then high pressure frozen and freeze substituted. Guinea pig organ of Corti was chosen as a test tissue because of the relative ease of dissection. After fixation, the samples were assessed for the quality of cellular preservation and the presence of artefacts resulting from the chemical fixation. Several cryo-protectants including hexadecene and the penetrating cryo-protectant glycerol were tested. As the tissue had already been stabilised by fixation it was considered that the effects of a penetrating cryoprotectant may be less deleterious and that penetration of the tissue may offer additional cryoprotectant benefit. However, these cryoprotectants produced variable preservation. Consistent preservation of large tissue samples was achieved using a yeast paste cryoprotectant (Fig. 5a–c). Preservation comparable to that of high pressure freezing alone was seen both in hair cells and in supporting cells, which showed smooth, non-granulated cytoplasm (Fig. 5, Fig. 6a–c) and well preserved nuclei (Fig. 5d). In the basal portion of IHCs, the synapse and surrounding structures were particularly well preserved by the use of the yeast-paste cryoprotectant (Fig. 5f). In samples frozen in the presence of glycerol, large gaps between the IHC membrane and the membrane of the neuronal terminal suggested some shrinkage of the terminals (Fig. 5e). In contrast the terminals of yeast-paste frozen samples did not show such gaps and exhibited a close apposition between the pre- and post-synaptic membrane along the length of the contact between the hair cell membrane and the synaptic terminal. In both cases synapses showed a consistent post-synaptic density and evidence of structure within the synapse.

In the apical portion hair cells fine structures of the cuticular plate, stereociliary rootlets, fibrillar connections and microtubules were well defined, as were microtubules in the apical cytoplasm (Fig. 6a). Microtubule bundles were also well preserved both in hair cells and in supporting cells, including the presence of structures between individual microtubules in the IHC (Fig. 6b and c).

The aim of the pre-fixation technique was to improve the preservation of the stereociliary actin bundle, which had proved difficult to preserve reliably by HPF alone. As with cellular preservation, the preservation of stereociliary actin was assessed in samples frozen in a variety of cryoprotectants. Pre-fixation dramatically improved the preservation of actin bundles in stereocilia in all the samples, and made the preservation of actin structure consistent. As with cellular preservation, yeast paste produced the most consistent preservation of actin structure (data not shown). Parallel actin, smooth stereociliary membranes and good preservation of the cuticular plate without granularity could all be observed (Fig. 6d). Tip links were also observed (inset panel in Fig. 6d). Parallel actin was shown in both longitudinal and transverse sections (Fig. 6d–f). In high magnification images of cross sections of stereocilia, the organisation of the actin filaments were clearly defined, similar to that seen in slam frozen samples (Fig. 4a), and closely parallel (Fig. 6e). At high magnifications, crosslinks between actin filaments were resolved (Fig. 6f arrows).

## 4 Discussion

As the evolution of techniques and instrumentation enhance the potential of electron microscopy, the preservation of tissue with minimal artefact formation becomes ever more important. In particular, use of electron tomography to determine three-dimensional structures demands that the entirety of the tissue under investigation is well preserved. As well as structural preservation, HPF techniques are also often used for the improved preservation of antigenicity. As fixation can be achieved without the use of chemical fixatives, the deleterious effects of chemical crosslinking on protein structure and configurations are avoided. Testing the effect of HPF on the antigenicity of inner ear tissues was beyond the scope of this study, and may require further modifications to the preservation procedure, for example removing the low temperature osmication that occurs during FS. Previous work in tissue, for example in *Caenorhabditis elegans*, has shown that lower antibody concentrations and polyclonal antiserum that could not be used in conventionally fixed or HPF preserved osmicated tissue could be used successfully in HPF preserved tissue freeze substituted without osmication (Claeys et al., 2004). Although the pre-fixation HPF method discussed here may not be suitable for immunogenicity studies, due to the use of glutaraldehyde, it is possible that a similar method with an alternative pre-fixative may be used.

Cryo-preservation has become a routine technique in high-resolution electron microscopy, and yet its use for preservation of inner ear tissue has been limited, perhaps by the perceived technical challenges of preserving large and structurally complex tissues such as the cochlea and vestibular organs. This work has shown that routine HPF/FS techniques can be used for the preservation of inner ear tissues, and demonstrated the advantages of a modified fixation technique for the preservation of stereocilia.

Routine HPF/FS protocols produced good preservation of inner ear tissue, with relatively few freezing artefacts. Preservation of cellular structures and organelles was good and our work combined with that of Meyer et al. (2009) suggests that rapid freezing can be utilised for the preservation of synaptic structures without obvious morphological changes to the tissue such as swelling or shrinkage of peripheral processes in mammalian and non-mammalian tissue. Pre-fixation HPF also produced excellent preservation of the synaptic structures and may be of great utility where large stretches of consistent preservation across the organ of Corti are required.

However, the preservation of stereociliary actin bundles proved to be problematic. The ‘tangling’ of the actin filaments observed suggested that the filaments and the cross-links between them were being damaged or distorted by the freezing process. Damage was shown in both HPF and slam-freezing preparations. Previous reports of slam-freezing preparation of stereocilia have indicated that only tissue 10–15 μm from the freezing front contained optimally frozen material (Kachar et al., 2000), and this distance is often shorter than the length of the stereocilia. In addition the impact often deforms the bundles, changing the shape of stereocilia and the arrangement of features such as interstereociliary links and tip links. Also, exposing hair cells for slam freezing requires the disruptive removal of overlying extracellular matrix (Kachar et al., 1990). In contrast, HPF preparations produced large depths of well-frozen tissue, often greater than 100 μm and did not require the removal of extracellular components to expose hair cells. HPF therefore, is a useful rapid freezing technique where cells need to be examined in context with the cells around them, for example hair cells and supporting cells, or where cells cannot be easily isolated from the surrounding structures.

The occasional well preserved stereocilia in HPF and the apparent gradient of preservation in slam freezing suggest that stereocilia actin bundles exhibit unusual freezing properties, and may be susceptible to small variations in the freezing rate during the freezing process. The observed peculiarities seemed to be confined to the parallel actin filament bundles; where observed, the microtubules of the kinocilium in the same hair bundles appeared unaffected (data not shown) and the cellular preservation, including that of the cuticular plate, in the same region was very good. There are several factors that could account for these properties: the structure of the stereociliary actin bundle itself; the forces exerted on it during the freezing process; and the treatment of the samples after freezing.

The actin bundle in a stereocilium is a paracrystalline array of F-actin filaments, cross-linked by proteins, similar to the packed actin filaments found in microvilli (Flock and Cheung, 1977, Derosier et al., 1980). In an ordered structure such as the stereociliary actin bundle distortions resulting from the growth of ice crystals may be more obvious than were they to occur in a less strictly ordered structure. Therefore the observed changes to actin structure may be partly explained by disruptions to the tissue becoming more apparent when the structure is highly ordered. It is also possible that the ordered structure of the actin bundles in stereocilia may affect the formation of ice crystals in the actin lattice. However, there is little evidence that water in biological structures behaves differently in terms of ice crystal formation compared to bulk water (Dubochet, 2007). F-actin lattices have also been successfully high pressure frozen in the past, both in isolated forms and within microvilli (Resch et al., 2002, Ohta et al., 2012).

The high pressure exerted on the tissue is another possible explanation for the disruption to stereociliary actin during HPF. Previous studies on the effects of pressure in HPF freezing have suggested, perhaps surprisingly, that few changes due to pressure are observed in frozen samples (Dubochet, 2007), although it has been suggested that chromatin and some phospholipid membranes may exhibit pressure related changes (Leforestier et al., 1996, Semmler et al., 1998). The previous successful preparation of F-actin bundles by HPF (Ohta et al., 2012) would argue against a pressure related effect on actin structure. This argument is supported by the observation that the “tangle” artefact was also present in samples “slam” frozen at ambient pressure.

After freezing, samples were freeze-substituted at −90 °C in acetone solutions containing stains and fixatives. The potential for re-crystallisation of ice in FS is still debated. The temperature at which FS occurs is theoretically low enough to prevent the re-crystallisation of ice in biological samples (Steinbrecht, 1985). It has been suggested that cubic ice (an alternative form of crystalline ice) may undergo a transition to hexagonal ice at −80 °C, but also that such devitrification events are unlikely to have significant effects on preservation, because molecules in the sample are almost immobile. Therefore ice damage to the sample is much more likely to occur during the freezing event (Dubochet, 2007). Water present in the sample during warming could also account for the damage, but the lengthy substitution times used in the experiments make this unlikely. It has recently been shown that in many tissues, including actin bundles of microvilli in *C. elegans* FS can be carried out in ninety minutes (McDonald and Webb, 2011).

The damage to the stereocilia actin is most likely an indication of failure to fully vitrify the tissue during HPF, but may be affected by other factors in the freezing and substitution processes. To separate these factors samples would have to be examined in frozen-hydrated sections, by a technique such as cryo-electron microscopy of vitreous sections (CEMOVIS) (Al-Amoudi et al., 2004) or cryo focused ion beam milling (cryo-FIB) (Lucic et al., 2013) both of which allow the thinning of frozen hydrated tissues so they may be directly examined in a transmission electron microscope. This would allow examination of the type of ice within distorted stereocilia, which would show if the ice had crystalline structure, indicating a freezing defect, or was vitreous, showing the effect was due to pressure or another factor. If the stereocilia were not distorted, this would indicate the post freezing handling of the samples was responsible. However such techniques are technically demanding, and it was not possible to carry out these experiments as part of this study.

Pre-fixation before HPF has been described several times as a technique for the handling of delicate or highly labile tissues, such as retina, nerves and virus infected DL1 insect cells. It was demonstrated that the hybrid technique, combined with the correct choice of cryo-protectant, could produce structural preservation superior to conventional fixation and close to that produced by HPF alone (Meissner and Schwarz, 1990, Sosinsky et al., 2005, Sosinsky et al., 2008). The work presented here shows that pre-fixation HPF applied to inner ear tissues also produces results superior to those often achieved by conventional fixation in both the preservation of the actin bundle and the smoothness of the stereociliary membranes. The preservation of cellular structure is close to that achieved with HPF alone. Most importantly, pre-fixation before HPF resulted in consistent preservation of the stereocilia actin bundle across the tissue sample. It may be pertinent to note in this context the work of Hirokawa and Tilney (1982) who examined actin in stereocilia of the chick basilar papillae by deep etching after freeze-fracture in samples that had been slam frozen either directly after isolation from cochlea (fresh frozen) or after glutaraldehyde fixation. The authors observed a difference between fixed and unfixed sample in the distance between the actin bundle and stereociliary membrane. They attributed this difference to the exposure of unfixed stereocilia to a potassium rich fluid (Hirokawa and Tilney, 1982). However, there do appear to be differences between the actin bundles in the two images, and a possible alternative explanation would be freezing induced changes to the actin bundle in the unfixed sample similar to that observed here.

The difference between cryo-protectants between pre-fixed HPF samples is puzzling. Pre-fixed tissue samples frozen with glycerol and 1-hexadecene both contained artefacts often associated with chemical fixation, including granularity of the cytoplasm and nuclei and shrinkage of the tissue but artefacts did not occur in samples frozen with yeast paste. In the case of glycerol deleterious effects may occur due to penetration of the cryoprotectant into the tissue, and as unfixed samples were not frozen with glycerol it is difficult to say where in the process problems may be occurring. However, no obvious differences were noticed between yeast paste and 1-hexadecene frozen samples in rapidly frozen samples without pre-fixation, making it likely that that the problem with this cryoprotecant is not occurring during HPF.

One possible explanation for this phenomenon would be a difference in the behaviour of the samples during the FS process. It is known that at low temperatures acetone does not dissolve 1-hexadecene and although efforts were made to remove any residue from around the samples, remaining 1-hexadecene may have impeded substitution. Glutaraldehyde improperly washed out of the tissue may have caused post-fixation artefacts and reacted with any unreacted osmium tetroxide in the warming tissue. Interactions like this illustrate the importance of the post-freezing processing of the tissue in structural preservation.

Work by Small (1981) has shown that the ordered structure of actin in lamellopodia can be well preserved by glutaraldehyde fixation, but was destroyed by the subsequent steps in conventional transmission electron microscopy processing, specifically post-fixation osmication of the sample and dehydration. It was also shown that the damage caused by osmication could be prevented by exposing the samples to smaller concentrations (0.2%) at a lower temperature (0 °C instead of room temperature) (Small, 1981). Cross-linked actin bundles appear to be somewhat more resistant to these procedures than individual filaments (Tilney et al., 1998, Small et al., 1999). However, the improvement of preservation of stereocilia in the pre-fixed HPF samples compared to conventional preparations is most likely the result of performing osmication and dehydration procedures at very low temperatures, as part of the FS process. The effects of room temperature dehydration in terms of water extraction and tissue shrinkage in animal and plant tissues have been well described (Boyde and Boyde, 1980). In FS, because substitution occurs below the temperature at which most water would be extracted from the sample, the artefacts caused by dehydration are minimal (Muller, 1988, Meissner and Schwarz, 1990).

Pre-fixation has the additional advantage of decreasing the time between the removal of inner ear tissue and the beginning of fixation. Although HPF will fix tissue within milliseconds, delicate dissection is required to produce tissue samples suitable for HPF fixation. These samples must be both small enough to freeze effectively, and free of any bone or calcified material that may impair later processing. Therefore, the time between sacrifice and freezing of inner ear tissues can be twenty minutes or longer. With the pre-fixation protocol, fine dissection can be carried out in the fixed tissue, reducing the potential for artefacts from dissection and deterioration of the tissue.

### 4.1 Conclusions

HPF of inner ear tissue using routine protocols will produce good preservation of tissue, and allows fixation of large depths of tissue by rapid freezing. However, the preservation of stereocilia by rapid-freezing processes is inconsistent, and therefore HPF alone is not suitable where large tissue depths are required, but preservation of stereocilia structure is also important. With careful selection of cryoprotectant, freezing and substituting procedure, pre-fixation HPF can give cellular preservation close to that of HPF alone and consistently preserve stereociliary actin, without the potential changes to actin bundle and dimensional changes to the stereocilia caused by room temperature dehydration in conventional fixation.

## Author contributions

Experimental work: AB, RT, BK, AF.

Article Preparation: AB, BK, CM, RF, AF.

All Authors have approved submission of this article.
