# Supplementary material for: Inner ear tissue preservation by rapid freezing: Improving fixation by high-pressure freezing and hybrid methods
Source: Hear Res. 2014 Sep;315:49–60. doi: 10.1016/j.heares.2014.06.006 (PMC4152001; doi:10.1016/j.heares.2014.06.006)
Supplement: Supplementary file 1 [file mmc1.docx]

**Table 1: Sample preparation protocols for each figure panel**

| **Figure Panels** | **Species** | **Fixation Method** | **Pre-fixation method** | **Cryo-protectant** | **Freeze substitution method** |
| --- | --- | --- | --- | --- | --- |
| 1A, B, F | Mouse | HPF | N/A | Dextran | 1 |
| 1C, D | Mouse | Conventional | N/A | N/A | N/A |
| 1E, G | Guinea Pig | HPF | N/A | Yeast paste | 1 |
| 1H | Guinea Pig | HPF | N/A | Hexadecene | 1 |
| 1I, J | Gerbil | HPF | N/A | Dextran | 1 |
| 2A, B | Newt | Conventional | N/A | N/A | N/A |
| 2C, D | Newt | HPF | N/A | Dextran | 1 |
| 3A | Mouse | HPF | N/A | Hexadecene | 1 |
| 3B, D | Mouse | HPF | N/A | Dextran | 1 |
| 3C | Gerbil | HPF | N/A | Dextran | 1 |
| 3E | Guinea Pig | HPF | N/A | Yeast paste | 1 |
| 4A | Rat | Slam-Freezing | N/A | N/A | 2 |
| 4B, C | Guinea Pig | Slam-Freezing | N/A | N/A | 1 |
| 5A, D, F | Guinea Pig | Pre-fixation HPF | Glutaraldehyde | Yeast paste | 3 |
| 5B, E | Guinea Pig | Pre-fixation HPF | Glutaraldehyde | Glycerol | 3 |
| 5C | Guinea Pig | Pre-fixation HPF | Glutaraldehyde | Hexadecene | 3 |
| 6A, B, D, E, F | Guinea Pig | Pre-fixation HPF | Glutaraldehyde | Yeast paste | 3 |
| 6C | Guinea Pig | Pre-fixation HPF | Glutaraldehyde | Hexadecene | 3 |
