# Supplementary material for: Inner ear tissue preservation by rapid freezing: Improving fixation by high-pressure freezing and hybrid methods
Source: Hear Res. 2014 Sep;315:49–60. doi: 10.1016/j.heares.2014.06.006 (PMC4152001; doi:10.1016/j.heares.2014.06.006)
Supplement: Supplementary file 2 [file mmc2.docx]

**Table 2: Freeze substitution methods**

| **1** | Temperature: -90°C  Medium: Acetone  Solutions:   - 0.1% tannic acid, 0.5% glutaraldehyde - 0.1% osmium tetroxide 1% uranyl acetate |
| --- | --- |
| **2** | Temperature: -90°C  Medium: Methanol  Solutions:   - 1.5% uranyl acetate |
| **3** | Temperature: -90°C  Medium: Acetone  Solutions:   - 0.1% tannic acid - 0.1% osmium tetroxide 1% uranyl acetate |
